# Genome of the Psychrophilic Bacterium *Bacillus psychrosaccharolyticus*, a Potential Source of 2′-Deoxyribosyltransferase for Industrial Nucleoside Synthesis

**DOI:** 10.1128/genomeA.00309-13

**Published:** 2013-05-30

**Authors:** Alba Fresco-Taboada, Carlos del Cerro, Jesús Fernández-Lucas, Miguel Arroyo, Carmen Acebal, José L. García, Isabel de la Mata

**Affiliations:** Department of Biochemistry and Molecular Biology, Facultad de Biología, Universidad Complutense de Madrid, Madrid, Spaina; Department of Environmental Biology, Centro de Investigaciones Biológicas, Consejo Superior de Investigaciones Científicas, Madrid, Spainb

## Abstract

Here we report the draft genome sequence of *Bacillus psychrosaccharolyticus*, a cold-adapted bacterium with biotechnological interest. The genome contains genes related to the ability of this microorganism to grow at low temperatures and includes a nucleoside 2′-deoxyribosyltransferase, which can be used in the industrial synthesis of modified nucleosides with therapeutic activity.

## GENOME ANNOUNCEMENT

*Bacillus psychrosaccharolyticus* (CECT 4074, ATCC 23296, DSM 6) is a facultative anaerobic Gram-positive psychrophilic bacterium, which can be found in soil and lowland marshes (1). This microorganism should be considered a psychrotrophic bacterium (2), because it grows well at temperatures close to 0°C and it is unable to grow at temperatures above 30°C, but it achieves the highest growth rate at 20°C.

Although several genome sequences from three psychrophilic bacteria (3–5), as well as two cold-adapted *Archaea* (6), have been recently published, no genome sequences of psychrotrophic bacilli have been reported so far. Availability of genome sequences of psychrophilic microorganisms is considered a valuable tool to elucidate cold-adaptive and other stress-adaptive mechanisms. Likewise, it is useful to search novel cold-adapted microbial enzymes with biotechnological interest, since these biocatalysts are more productive at low temperatures than their mesophilic or thermophilic counterparts (7, 8). In this sense, a nucleoside 2′-deoxyribosyltransferase activity has been described in *B. psychrosaccharolyticus* CECT 4074 (9), but the enzyme responsible for such a reaction has never been isolated. Such enzymatic reactions show biotechnological interest since the enzyme (nucleoside deoxyribosyltransferase [NDT], EC 2.4.2.6) catalyzes the interchange of bases between deoxyribonucleosides, allowing the stereo- and regioselective synthesis of natural and nonnatural nucleoside analogues under mild conditions (10). This is an interesting alternative to traditional chemical procedures for the synthesis of these modified nucleosides (11) which are extensively used as antiviral and anticancer agents (12, 13). Thus, the availability of new nucleoside 2′-deoxyribosyltransferases with improved activity and stability and/or novel specificity could expand the application of these enzymes for the preparation of modified nucleosides in the pharmaceutical industry.

The draft genome sequence of *B. psychrosaccharolyticus* CECT 4074 was obtained from a shotgun library constructed and sequenced using a titanium kit in a GS-FLX instrument (Roche Diagnostics, Banford, CT) at the Fundación Parque Científico de Madrid (Spain). A total of 2.2 million reads (19.9-fold coverage) were first preliminarily assembled by Newbler 2.5.3 software, yielding 405 large contigs. To improve the draft quality, additional sequencing was carried out using Illumina Miseq at the Unidad de Genómica Cantoblanco. In this case, a total of 5.29 million reads (168-fold coverage) were manually assembled, reducing the number of large contigs to 265. The open reading frames (ORFs) and RNA genes were predicted by the RAST server (14). The genome of 4.6 Mbp in size has 38.7% G+C content and carries 6,201 predicted ORFs (4,741 ORFs have putative assigned functions), 15 rRNAs, and 85 tRNAs. *B. psychrosaccharolyticus* contains the Embden-Meyerhof and tricarboxylic acid (TCA) cycle pathways as well as the enzymes for the glyoxylate cycle. In addition, the genome includes genes encoding five different types of translation elongation factors (G, Tu, Ts, LepA, and P), which might be related to the ability of this microorganism to grow at low temperatures (15). Remarkably, CspA is the unique cold-shock protein found in this genome (3, 16). Finally, the sequence of the gene encoding the NDT enzyme was identified in the genome, therefore confirming *B. psychrosaccharolyticus* as a microbial source for novel psychrophilic enzymes with potential biotechnological applications.

### Nucleotide sequence accession numbers.

This whole-genome shotgun project has been deposited at DDBJ/EMBL/GenBank under the accession number AJTN00000000. The version described in this paper is the second version, AJTN02000000.
